# Editorial for the Special Issue: “Monitoring of Behavior, Affective States, and Health to Identify Welfare Concerns of Farm Animals”

**DOI:** 10.3390/ani15162331

**Published:** 2025-08-08

**Authors:** Helen Louton

**Affiliations:** Chair of Animal Welfare, Ethology, Animal Hygiene and Animal Husbandry, Department of Veterinary Sciences, Faculty of Veterinary Medicine, LMU Munich, Veterinärstraße 13/R, D-80539 München, Germany; h.louton@lmu.de

Ensuring the welfare of farm animals remains a core objective in contemporary animal husbandry. This Special Issue of *Animals*, titled “Monitoring of Behavior, Affective States, and Health to Identify Welfare Concerns of Farm Animals,” brings together 13 insightful contributions that explore innovative and science-based approaches to assess animal welfare objectively and reliably.

The articles in this Special Issue focus on various methodologies to monitor the behavior, affective states, and health of livestock. A central theme is the development of measurable, farm-independent, scientifically grounded, and reproducible indicators that can identify welfare concerns early and accurately. These indicators are intended not only to reflect the current state of animal well-being but also to serve as early warning signs of potential issues.

Several studies emphasize the application of sensor technologies and automated monitoring systems that allow for continuous, real-time observation of behavioral and physiological parameters. These technologies facilitate the early detection of subtle changes in animal condition and support timely interventions.

Other contributions delve into the assessment of affective states—that is, the emotional experiences of animals. The research presented here introduces methods to identify and quantify both positive and negative emotional states, thereby advancing a more comprehensive understanding of animal welfare.

Collectively, the studies featured in this Special Issue provide valuable insights and practical tools for farmers, veterinarians, researchers, and policymakers. They mark significant progress in the development of robust animal welfare indicators and monitoring systems that can enhance animal care and ethical standards across livestock production systems.

This Special Issue underscores that the integration of modern technologies with science-driven welfare assessment strategies is a promising path forward to sustainably improve animal well-being.

